# Impact of digital video analytics on accuracy of chemobehavioural phenotyping in aquatic toxicology

**DOI:** 10.7717/peerj.7367

**Published:** 2019-08-05

**Authors:** Jason Henry, Alvaro Rodriguez, Donald Wlodkowic

**Affiliations:** 1 School of Science, RMIT University, Melbourne, VIC, Australia; 2 Biomedical Research Institute A Coruña (INIBIC), University Hospital Complex of A Coruña, Coruña, Spain; 3 Department of Computer Science, University of A Coruña, Spain

**Keywords:** Animal, Behaviour, Tracking, Video, Toxicity, Phenomics

## Abstract

Chemobehavioural phenotypic analysis using small aquatic model organisms is becoming an important toolbox in aquatic ecotoxicology and neuroactive drug discovery. The analysis of the organisms’ behavior is usually performed by combining digital video recording with animal tracking software. This software detects the organisms in the video frames, and reconstructs their movement trajectory using image processing algorithms. In this work we investigated the impact of video file characteristics, video optimization techniques and differences in animal tracking algorithms on the accuracy of quantitative neurobehavioural endpoints. We employed larval stages of a free-swimming euryhaline crustacean *Artemia franciscana*,commonly used for marine ecotoxicity testing, as a proxy modelto assess the effects of video analytics on quantitative behavioural parameters. We evaluated parameters such as data processing speed, tracking precision, capability to perform high-throughput batch processing of video files. Using a model toxicant the software algorithms were also finally benchmarked against one another. Our data indicates that variability in video file parameters; such as resolution, frame rate, file containers types, codecs and compression levels, can be a source of experimental biases in behavioural analysis. Similarly, the variability in data outputs between different tracking algorithms should be taken into account when designing standardized behavioral experiments and conducting chemobehavioural phenotyping.

## Introduction

The acquisition of organism-wide high-dimensional phenotypic data (phenomics) has undergone an evolution to existing molecular omics paradigms over the last decade (Houle, Govindaraju & Omholt, 2010). This evolution is leading to advances in environmental toxicology and neoclassical phenotypic drug discovery techniques (Faimali et al., 2006; Hellou, 2011; Kokel & Peterson, 2008; Zhang et al., 2019). Behaviour is one of the sub-lethal endpoints that can change temporally and relative to acute and chronic exposure to chemical agents (Chapman, 2007; Gerhardt, 2007; Hellou, 2011). Chemobehavioural phenotyping embraces the complexity of multicellular organisms thus reflecting integrated functional changes at highest levels of organismal organization (Zhang et al., 2019). Manifestations of behavioural phenotypes can transcend multiple levels of biological structure and function; providing causative links between genetic, subcellular and physiological processes (Chapman, 2007; Gerhardt, 2007; Hellou, 2011). This can deliver an overarching strategy to identify functional effects of chemicals with particular biological and/or ecological effects (Faimali et al., 2017; Zhang et al., 2019). The analysis of diverse behavioural endpoints such as locomotory activity, non-directional and directional responses to chemical stimuli, predator-prey interactions, feeding rate, mating rituals and 3D spatial orientation have been reported in behavioural ecotoxicology (Bownik, 2017; Chapman, 2007; Gerhardt, 2007; Hellou, 2011; Pyle & Ford, 2017). It is also postulated that high-throughput chemobehavioural phenomics using simplified digital fingerprints of behaviors can become an important toolbox for discovery of novel neuroactive medicines, screening for neurotoxic effects as well as identify specific nerve poisons and their antidotes (Bruni, Lakhani & Kokel, 2014; Kokel & Peterson, 2008).

Behavioral parameters are predominately analyzed using video recording and video processing techniques with a dedicated animal tracking software suite (Green et al., 2012; Noldus, Spink & Tegelenbosch, 2001; Rodriguez et al., 2017). Analog video tracking technologies were introduced in the late 1980s and provided significant advantages in terms of speed of data acquisition and accuracy over the previous manual counting methods and non-imaging analytical devices (Green et al., 2012; Noldus, Spink & Tegelenbosch, 2001). In the 21st century, many disciplines of bioscience have been utilizing low-cost digital video recording to conduct behavioural studies (Bownik, 2017; Huang, Campana & Wlodkowic, 2017; Huang et al., 2016). Chemobehavioural phenotypic analysis has been rejuvenated with an increase in user-friendly automated acquisition systems and animal tracking suites to measure behavioural endpoints (Franco-Restrepo, Forero & Vargas, 2019; Green et al., 2012; Noldus, Spink & Tegelenbosch, 2001; Rodriguez et al., 2018).

Digital video-based tracking of animal behavior involves deconvolution of pattern analysis in a grid of pixels on individual frames of video file. Software algorithms analyze each frame of the video file to distinguish the tracked animals from the background (Franco-Restrepo, Forero & Vargas, 2019; Noldus, Spink & Tegelenbosch, 2001; Rodriguez et al., 2018). This is performed on the basis of semi-automated adjustment of a pixel intensity threshold and color saturation values. Each detected object is then assigned a mathematical center of gravity (centroid) that is derived from the average surface area (Franco-Restrepo, Forero & Vargas, 2019; Noldus, Spink & Tegelenbosch, 2001; Rodriguez et al., 2018). Automatic frame-by-frame tracking leads to time-stamped x-y coordinate pairs assigned to centroids of each detected objects and provides the foundation to reconstruct animal trajectories and estimate occupancy heatmaps (Noldus, Spink & Tegelenbosch, 2001).

The time-stamped x-y coordinates assigned to centroids of each detected object are used for calculations of quantitative behavioural data sets such as; average speed of locomotion, average distance travelled of an animal or the distance between several individually identified animals (Franco-Restrepo, Forero & Vargas, 2019; Noldus, Spink & Tegelenbosch, 2001; Rodriguez et al., 2018).

Despite the increasing number of reports using digital video analytics in chemobehavioral studies there is a paucity of data on how video file characteristics and various animal-tracking algorithms affect accuracy and reproducibility of neurobehavioural endpoints (Wang et al., 2017a). For instance, it is unknown if outcomes of behavioural analysis will differ when the source data are acquired using different digital camera settings and/or analyzed using different algorithms. In this regard many researchers utilizing modern digital camera recorders are not entirely familiar with a surprisingly large number of variables that need to be considered. Video files are not only characterized by their resolution (number of pixels per area of frame), but are in fact a series of still frames recorded in a unit of time (Bernas, 2005; Boudier & Shotton, 1999; Cerina, Iozzia & Mainardi, 2019; Wallace et al., 2018; Wang et al., 2017a). As such, each video file can be recorded at a different frame rate usually denoted in frames per second (fps). Since a sequence of high-resolution frames is usually memory intensive, video data is often compressed using a range of available software codecs (Cerina, Iozzia & Mainardi, 2019; Rapczynski, Werner & Al-Hamadi, 2019). The compressed data streams can conform to very different standards and codec selection and their chosen settings can impact the process of animal tracking (Boudier & Shotton, 1999). This is because video codecs vary significantly in their level of data preservation. For instance, heavy compression can generate small video files that are easier to store and compute but at a cost of significant amount of data being artificially interpolated (Boudier & Shotton, 1999; Cerina, Iozzia & Mainardi, 2019; Rapczynski, Werner & Al-Hamadi, 2019). Consequences of the above on quantitative behavioural end-points have not been extensively explored. Furthermore, little data exists on comparative analysis of video file optimization strategies, such as background subtraction techniques, on the performance and accuracy of animal tracking algorithms (Wang et al., 2017a; Wang et al., 2017b). Since the contrast-based detection of animals heavily depends on distinguishing them from the background, the use of background subtraction techniques can greatly accelerate semi-automated thresholding and increase overall accuracy of analysis (Rodriguez et al., 2017; Rodriguez et al., 2018). Lastly, there is relatively little experimental benchmarking data on the capability of animal tracking software to enable high-throughput parallel analysis of multiple samples and/or automated batch processing of video files (Green et al., 2012; McCarroll et al., 2016).

*Artemia franciscana* was used as a proxy model for chemobehavioral analysis because of their small size and relatively fast, continuous locomotory activities. Such behavioural characteristics parameters are challenging for the video-based animal tracking algorithms especially when large number of animals are to be tracked simultaneously. Additional reasoning for the selection of this species was that larval stages of brine shrimp are commonly used and broadly accepted models in marine ecotoxicology. A considerable number of behavioural toxicity studies have been performed using *Artemia sp.* nauplii in recent years. However, so far none of the published studies considered a high-throughput behavioural chemophenotypic screening strategies.

The main motivation of this work was to investigate the impact of video file characteristics, video optimization techniques and inherent differences in animal tracking algorithms on the accuracy of quantitative neurobehavioural data. We also wanted to demonstrate a capacity to perform high-throughput automated data analysis of quantitative behavioural data in aquatic toxicology. In this regard we demonstrated rapid and inexpensive methods to improve the process of thresholding during contrast-based animal detection and tracking as well different data processing pipelines. Two existing mainstreams commercial as well as one open source software packages were evaluated with higher-throughput toxicological phenotyping to benchmark their associated algorithms. The secondary goal of this work was to guide the scientists in the variability of parameters that need to be considered when undertaking chemobehavioural studies.

## Materials and Methods

### Test organisms and exposures

Eggs of the marine crustacean *Artemia franciscana* (Southern Biological Pty Ltd, Melbourne, Australia) were hatched according to a standard operating protocol as described before (Huang et al., 2016). Briefly approximately 100 eggs were placed in an 85 mm Petri dish filled with approximately 35 mL of filtered sea water (pH 8.0 ± 0.5). The vessels were incubated at 25 °C and illuminated at 3000–4000 lux for 24 hours to induce hatching. Upon hatching, viable larval stages were manually sorted to achieve uniform developmental staging at first instar and moved to a fresh medium at 22 hours. Up to four naupli were manually aspirated and randomly loaded into each test chamber. The model toxicant organophosphate, Chlorpyrifos, tested was dissolved directly in sea water as it has good water solubility. Toxicity testing protocol adhered to international “OECD Guidelines for the Testing of Chemicals” (http://www.oecd.org/chemicalsafety/testing/oecdguidelinesforthetestingofchemicals.htm).

### Test chamber design and fabrication

All test chambers were designed using CorelDraw X3 (Corel Corporation, Ottawa, Ontario, Canada) CAD package as described before (Huang, Campana & Wlodkowic, 2017). The initial stages of testing for comparison of video setup utilised a test chamber with a diameter of 15.6 mm, a depth of three mm and a nominal volume of 574 µL. The validation stages of testing with toxicants involved reducing the test chamber to a diameter of 11 mm with a nominal volume of 285 µL. All vessels were fabricated in a fully biocompatible polymethyl methacrylate (PMMA) transparent thermoplastic using a non-contact 30W infrared laser machining system (Universal Laser Systems, Scottsdale, AZ, USA) as described before (Huang et al., 2016).

### Digital video recording

Behavioral data was acquired using a custom build digital video imaging system. The entire neurobehavioural analysis system was installed in a temperature-stabilized room (25 ± 0.5 °C) and consisted of a cold LED (5500 K, 820Lux) backlit illumination stage and a digital video camera mounted on a vibration-less photographic column (Polaroid M3, Polaroid Inc, USA). The digital imaging was realized by a Canon EOS 7D Mk2 system, equipped with a CMOS APS-C type sensor (22.4 × 15.0 mm, 20.20 Megapixel resolution, and individual pixel size of 4.1 µm). The camera was paired with a true 1:1 Macro lens with a focal length of 90 mm (Tamron Corp, Commack, NY, USA). The setup of the system enabled simultanous imaging of a large area consisting of up to 28 test chambers, thus eliminating any need for a motorized stage and potentially disruptive manipulations of the test specimens. For simplicity of data analysis a scaled down array of four test chambers was used for the majority of the software comparisons as a multitude of video files could be post-processed to represent further replicates. The video recording specifications were modified as required by the testing for each technical recording.

Native videos of 55 seconds in duration were recorded with a resolution of 1,920 × 1,080 pixels (1080 p) and a framerate of 24 fps. The final toxicity tests were filmed in a custom array of chambers (7 by 4 matrix) with three organisms loaded per test chamber. This setup was recorded every 6 h for up to 24 h. All video files were acquired using an external High-Definition Multimedia Interface (HDMI) recorder (Atomos Shogun, Melbourne, Australia) equiped with a programmable time-resolved video acquisition functionality. Native files were saved in .mov digital containers and encoded with a ProRes 422 HQ codec that provided no temporal compression artefacts (Inter frame-only encoding) and variable bitrate.

### Computational workstation

All video data processing and animal tracking was performed on a Dell Precision 7810 PC workstation designed for parallel video processing tasks. The latter had two six-core Intel Xeon processors (CPU E5−2620 V3 2.40GHz) with hyper-threading technology providing a total of 24 logic processors, 32 GB random access memory (RAM) and an AMD FirePro W5100 graphics card. The operating system was a 64 bit Windows 10 Pro.

### Video file transcoding

Native files were ingested into a DaVinci Resolve 15 (Blackmagic Design, Australia) video-editing software suite used for transcoding and file optimization. To compare the impact of different compression levels. The native sets of video files were transcoded using Uncompressed RGB 10-bit (low compression), Uncompressed YUV 10-bit (medium compression) and GoProCineForm YUV 10-bit (high compression) codecs to common .avi-type containers to allow for comparison between the different software suites. The selection of codecs was based on their compatibility with all the animal tracking software packages (see below) and the ability to achieve different levels of compression as shown in Table 1. To obtain identical video files of different resolution for comparative analysis, the native 1080 p files were transcoded using the GoPro Cineform YUV codec to obtain a down sampled range of resolutions such as 1,280 × 720 pixels (720 p) and 640 × 480 pixels (480 p). Similarly to obtain to obtain identical videos files with different frame rates the sets of native files recorded at 60 fps were transcoded using GoPro Cineform YUV codec to obtain files with 24, 30 and 50 fps.

**Table 1:** Characteristics of compression-decompression software (codec) used for transcoding of video files to obtain different levels of video data compression.

	**Compression**		
**File parameter**	**Low**	**Medium**	**High**
Codec	Uncompressed RGB 10-bit	Uncompressed YUV 10-bit	GoPro CineForm YUV 10-bit
Resolution	1080 p	1080 p	1080 p
Framerate	24 fps	24 fps	24 fps
Container	.avi	.avi	.avi
File Size	10200 mb	6810 mb	213 mb

### Video file optimization for animal tracking

Native video files were post-processed using a combination of Adobe Photoshop CC 2017 (Adobe, USA) and DaVinci Resolve 15 (Blackmagic Design, Australia) video editing software. The optimization techniques included digital cropping, sharpening and background subtraction. The latter involved initially creating a digital background mask in Adobe Photoshop across the test wells. The digital mask could be completely white or black for positive or negative background subtraction respectively. Digital masks were automatically exported as a .png file types for every video file.

The native video files were imported into DaVinci Resolve 15 video editing suite along with the respective digital background masks. For each video file, a single freeze frame was initially generated and subtracted from the entire time period of the video along with the associate digital mask using the inspector composite mode feature. This technique resulted in a video with perfectly black or white background in direct contrast to the organism, all manipulations describe above were performed using DaVinci Resolve automated batch processing capabilities in a high throughput fashion.

### Video file analytics

For comparative analysis identical video file sets were analysed with three behavioural analysis software packages: Ethovision XT ver. 14 (Noldus Information Technology, The Netherlands) (Marechal et al., 2004; Noldus, Spink & Tegelenbosch, 2001; Spink et al., 2001), ToxTrac ver 2.83 (open source software freely available at sourceforge.net/projects/toxtrac) (Rodriguez et al., 2017; Rodriguez et al., 2018), and LoliTrack ver. 4 (Loligo^^®^^ Systems, Viborg, Denmark) (Table 2). All software suites selected for this study employed a principle of contrast detection to identify subjects on video files. For this to occur any test animal to be tracked must be placed against a contrasting background. Software employed in this study were also capable of creating unique virtual zones of interests on video files where the proportion of time spent by the animals in those regions as well as behavioral parameters can also be analyzed (Table 2).

**Table 2:** Features of animal tracking software suites used in the comparative benchmarking.

**Software Suite**	**Ethovision**	**Toxtrac**	**Lolitrack**
*Data processing time*			
Native video	Medium	Slow	Slow
Positive background subtraction	Fast	Fast	Medium
Negative background subtraction	Fast	Fast	Medium
*Perceived user interface experience*			
File import	Medium	Fast	Slow
Arena definition	Fast	Medium	Medium
Subject detection	Fast	Medium	Slow
Subject tracking	Fast	Fast	Slow
Data acquisition	Fast	Fast	Slow
Batch processing	Fast	Medium	N/A
Data export	Fast	Medium	Slow
*Quantitative data outputs*			
Pixel-to-distance calibration	Yes	Yes	Yes
Numerical data outputs	Yes	Yes	Yes
Zone analysis	Yes	Yes	Yes
Population averages	No	Yes	No
*Graphical data outputs*			
Trajectory reconstruction	Yes	Yes	Yes
Exploration heatmaps	Yes	Yes	No

The data generated by analysis of each individual video was varied between software suites. Toxtrac provided averaged endpoint data for combined from all subjects in an arena for the duration of the video. Ethovision and LoliTrack provided averaged endpoint data for individual subjects in an arena for the duration of the video.

### Statistical analysis

Replicate data from different experiments was pooled. The behavioral data was averaged out as an average per unit of time (minute). Cumulative distance travelled by animals was measured by summing the incremental distance moved by each animal between frames of video files. Data was then averaged between individual animals kept in the same chamber. Statistical analysis and data presentation was performed in both Prism 8 (GraphPad Software Inc, San Diego, CA, USA) and RStudio V1.1.463 (RStudio Team, 2018) as before (Huang et al., 2018). Data sets were tested for normality of distribution, a one-way ANOVA was then used to test for significance. When significant exposure effects were found, a post-hoc Tukey’s test were performed to identify significance between treatment-related effects and time point with independently run control tests. Significance was set at *p* < 0.05.

## Results and Discussion

### Impact of video file compression on accuracy of animal tracking

**Figure 1:**
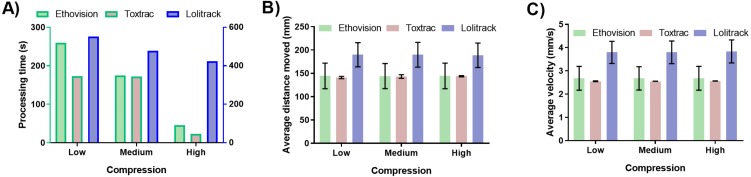
Impact of digital video data stream compression on (A) processing time of animal tracking, (B) computed average animal distance moved and (C) computed average animal velocity. To obtain low, medium and high compression ratios the identical sets of native video files were processed using Uncompressed RGB, Uncompressed YUV and GoPro CineForm YUV codecs, respectively.

For all tested animal-tracking algorithms the computing time was proportionally smaller with an increased digital compression levels (Fig. 1A, Table 1). The highest compression settings used generated files that were five times smaller than native ones and the processing time of the latter was also at least five times faster in Ethovision and ToxTrac (Fig. 1A). Lolitrack did not exhibit the same increase in speed of processing time despite the file size change. Interestingly accuracy of quantitative behavioral endpoints demonstrated here as computed average animal distance moved and average animal velocity showed negligible levels of variability between different levels of digital compression (Fig. 1B and Fig. 1C). Significant differences were, however, observed for processing time and accuracy of data between the algorithms employed. In this regard the LoliTrack software was at least nine times slower at high compression and up to three times slower at low and medium compression in computing the subject tracking. The end point quantitative data differed by up to approximately 30% as compared to Ethovision XT and ToxTrac (Fig. 1).

The data demonstrated that compression and thus size of video file plays an integral role in the high throughput neurobehavioural pipeline. Transcoding of native video files into a different codec can drastically reduce the processing time while maintaining the precision of the endpoint data. The results highlight also an inherent variability between the tracking algorithms. Of note, the .avi file format used here is at present considered to be an obsolete standard, disadvantaged by very large file sizes and limited selection of modern codecs. However, the selection of .avi codecs was necessitated by their compatibility with the all animal tracking software packages used in this study (Table 1). In this regard, the LoliTrack is the only software suite that limits the user to .avi files.

### Impact of video file resolution on accuracy of animal tracking

**Figure 2:**
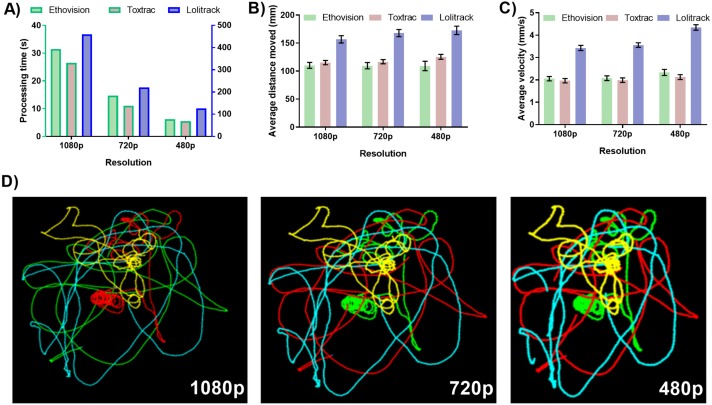
Impact of digital video file resolution on (A) processing time (B) computed average animal distance moved, (C) computed average animal velocity and (D) reconstruction of animal trajectories. To obtain 720p and 480p resolution the identical sets of native video files recorded at 1080p were transcoded using GoPro CineForm YUV codec. For simplicity of data presentation, the reconstructed trajectories were generated using ToxTrack ver 2.83. Note that the right axis represents data for Lolitrack software.

The down sampling of video file resolution resulted in proportionally faster computing times for all tested animal tracking software. The smallest tested resolution of 480 p returned computing time on average four times faster than the native resolution of 1080 p (Fig. 2A). The accuracy of quantitative the behavioral endpoints average animal distance travelled and average velocity showed negligible levels of variability between different levels of tested resolutions (Figs. 2B and 2C). However, LoliTrack software was again characterized by at least fifteen times slower computing time while its quantitative data outputs differed by at least approximately 40% as compared to Ethovision XT and ToxTrac (Fig. 2).

The resolution of a video file is integral when taking into account the small size of the tracked object. The measured pixel distance (calibrated to a nominal output in mm) for the movement of each organism in a well is only as accurate as the amount of pixels the organism can be thresholded (or measured) to move across. Thus the higher the resolution, the higher the accuracy of distance travelled and as a derivative also velocity and acceleration. When looking into the future one can expect automated higher throughput data acquisition system capable of simultaneously filming multiple test chambers. In such situations resolution of the camera sensor becomes a key factor requiring implementation of high definition or even ultra-high definition (4K and 8K) sensors. This is because with the larger the overall acquisition area the fewer of the pixels representing each test chamber will have assigned to threshold the organism against. To enable accurate thresholding ToxTrac recommends the subject size of an animal to be at least 50 pixels. Ethovision has stated that thresholding of subjects is possible with arena pixel quantity being 200 times larger than that of the target animal. The balancing of resolution to arena and organism ratio will be a key factor in future ultra-high throughput video recording systems. Unfortunately none of the animal tracking software available at present is capable of handling ultra-high definition video files. The results shown in Fig. 2 exhibit a consistency of results across the three tested resolutions. This provides key insights into the ratio described above. The quality and detail in the graphical data outputs of the trajectory and occupancy maps also improved in quality in ToxTrac with increase in resolution. Another key point to factor into this equation is the user time taken to correctly threshold the organism against the background in each software suite. When there is a high number of pixels per arena (high resolution), the user time taken to threshold the organism against the background is qualitatively reduced by 90% when comparing 1080 p to 480 p; this makes batch processing particularly easy. Overall both ToxTrac and Ethovision demonstrated a high precision in quantitative outputs in all resolutions.

### Impact of video frame rate on accuracy of animal tracking

**Figure 3:**
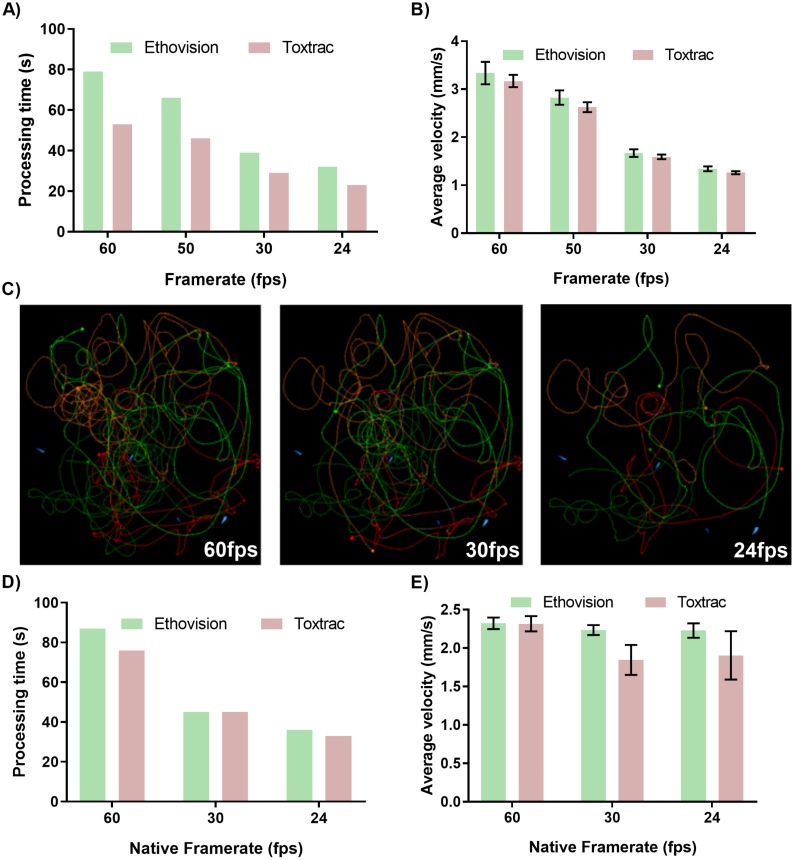
Impact of transcoded and native video file frame rates on average processing time of animal tracking (A, D) and computed parameter of average animal distance moved (B, E). Graphical representation of reconstructed animal trajectories (C) demonstrates significant loss of data when video files with transcoded frame rates are employed for animal tracking. To obtain transcoded files with 24, 30 and 60 fps the identical sets of native video files recorded at 60 fps were transcoded using GoPro CineForm YUV codec. For simplicity of data presentation the reconstructed trajectories presented in (C) were generated using only Ethovision XT ver. 14.

At first we evaluated impact of digital transcoding of native files recorded at 60 fps to lower frame rates that had a proportional impact on reduction of processing time (Fig. 3A). Accordingly, it took Ethovision XT algorithms approximately 80 and 30 s to compute animal tracking on a 55 s video clip at 60 fps vs. 24 fps, respectively (Fig. 3A). The impact of transcoded frame rates on accuracy of behavioral endpoints was however substantial. Transcoding of 60 fps to a 24 fps video led to on average 50% of the computed values for average animal velocity (Figs. 3B and 3C). The reconstruction of animal trajectories on transcoded files clearly demonstrates the loss of data during file processing to lower frame rate (Fig. 3C).

The analysis of video files at native frame rates (recorded in camera and not transcoded in video post-processing pipeline) depicted that lower fps values resulted in proportionally faster computing times for both tested animal tracking software (Fig. 3D). The accuracy of quantitative behavioral endpoints such as computed average velocity showed negligible levels of variability between 24, 30 and 60 fps for Ethovision XT (Fig. 3E). Interestingly, quantitative data outputs computed by the ToxTrac diverged from Ethovision at 24 and 30 fps by approximately 20% (Fig. 3E).

The above data demonstrate that frame rate of video file can have a major impact on both performance and accuracy of animal tracking algorithms. The results highlight that if the framerate is artificially downgraded in the post-processing pipeline to obtain a small file size, although no visual distortion can be seen, the tracking algorithms cannot interpolate the data lost during a transcoding process. This phenomenon can be easily visualised with the graphical trajectory reconstruction (Fig. 3C). The transcoding process lead to frames being completely removed from the video file, which results in data gaps that cannot be interpolated. Interestingly, it was found that using different native framerates pre-set at the data acquisition stage had negligible impact on the precision of the data output. It is important to note, however, that Ethovision is the only software capable of batch processing 60 fps video files. ToxTrac can only process one 60 fps video file at a time.

### Video file optimization techniques for improved animal tracking

**Figure 4:**
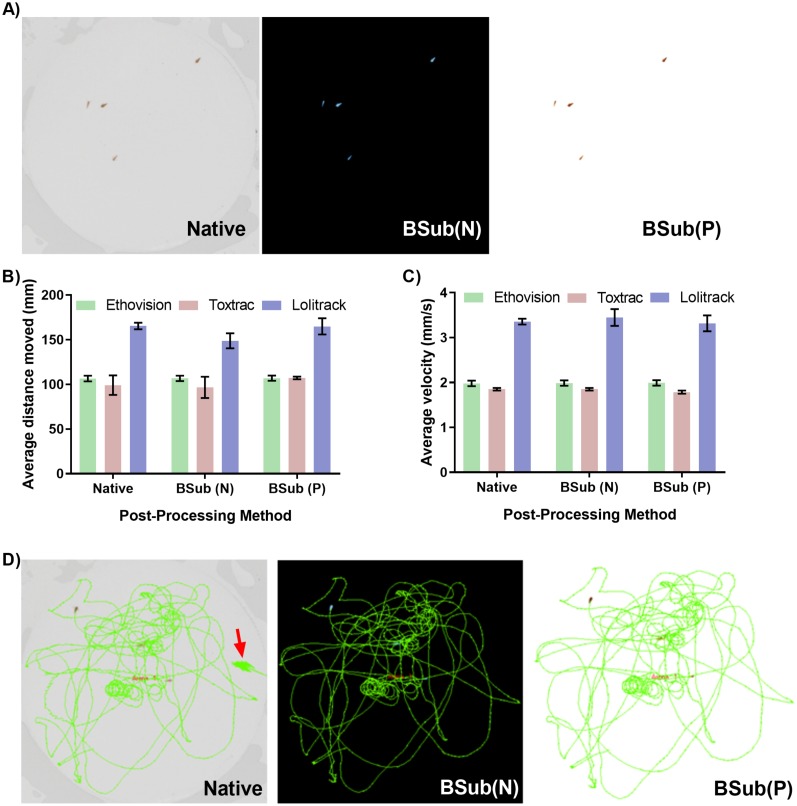
Impact of background subtraction techniques on accuracy of animal tracking and quantitative behavioural endpoints. (A) Demonstration of negative and positive background subtraction to improve accuracy and speed of thresholding and object detection. Background subtraction techniques do not significantly affect computed parameters of average animal distance moved (B), animal velocity (C) or graphical reconstructed of animal trajectories (D). For simplicity of data presentation the reconstructed trajectories presented in (D) were generated using only Lolitrack ver. 4. Note the minor discrepancies due to thresholding difficulties in processing the native video.

The contrast-based detection and tracking of objects on video files heavily relies on accurate distinguishing of them from the background. The potentially complex test chambers as well as uneven lighting conditions during experiments can introduce significant obstacles for reproducible video-based animal tracking. Therefore, efficient video file optimization strategies, such as background subtraction techniques that can be performed in high-throughput, aid in rapid thresholding. Figure 4A demonstrates a rapid optimization strategy utilizing both negative and positive background subtraction method applied in a freely available video editing software. The LoliTrack software was qualitatively considered the most difficult to perform effective thresholding of the objects and was thus used to benchmark the necessity for background subtraction techniques. The quantitative data outputs demonstrate consistent accuracy between the optimized and non-optimized video files (Figs. 4B–4D). However, again the user time required to set optimum threshold levels was qualitatively twice as fast when using optimized files; of high benefit to the batch processing pipeline. An additional process is included and therefore additional time taken in post-processing the video files to allow for this ease of thresholding; however as both softwares are capable of batch processing a large quantity of files this time is easily made up. Furthermore, as depicted in Fig. 4D, the accuracy of graphical trajectory reconstructions benefited from optimization techniques. Namely, despite substantial user time spent in correctly thresholding the native video files, small errors can still occur and potentially influence the output data (Fig. 4D).

We conclude that when analyzing hundreds of samples the manual optimization of threshold levels for every video file introduces unnecessary analytical bottlenecks. We postulate that to improve efficiency of analytical pipelines in behavioral studied the positive and/or negative background subtraction techniques can effectively be performed in freely available software suites that support automatically batch processing for increased throughput.

### High-throughput behavioural phenotyping–simultaneous tracking of multiple specimens

**Figure 5:**
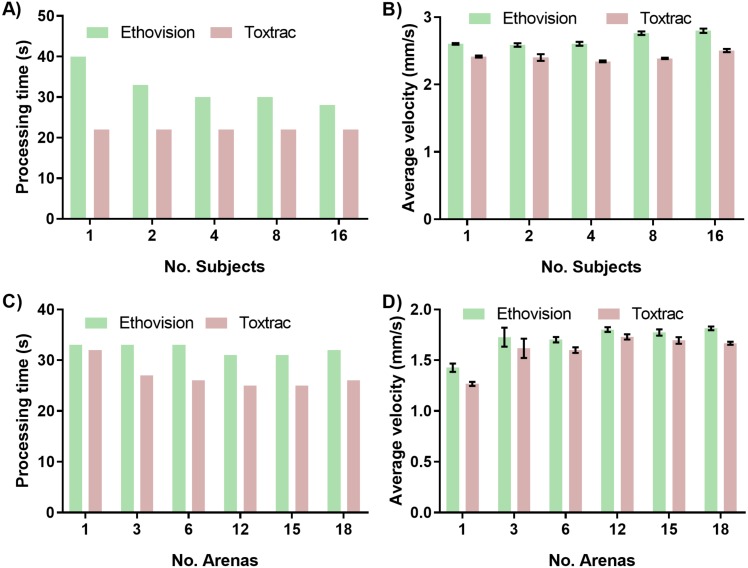
Impact of simultaneous tracking of multiple specimens on computing speed and accuracy of behavioural endpoints. The effects of increasing number of specimens tracked in one arena on (A) average processing time of animal tracking and (B) accuracy denoted as computed parameter of average animal velocity (B). The effects of altering the quantity of detection arenas on (C) average processing time of animal tracking and (D) computed endpoint parameter of average animal velocity.

One of the approaches to achieve higher throughput analysis in behavioral phenomics is simultaneous recording of multiple organisms in a single arena. The subsequent analysis of multiple objects can be challenging, as it requires continuous and reliable identification of all individual animals simultaneously. This means that although the software doesn’t necessarily lose track of a specific organism, the animal has the potential to cross paths close enough to another subject causing the two to switch identities in the eyes of the software (Rodriguez et al., 2017; Rodriguez et al., 2018). This is particularly important when quantitating the cognitive responses of an organism in a directional choice maze. To avoid this identity error Ethovision recommends keeping the number of organisms per arena to be less than 16 and ToxTrac between 10 and 20. This identity error was qualitatively observed in ToxTrac at more than 16 organisms per arena and as such this was the maximum organisms per arena that was tested for precision comparison. To assess this we first evaluated impact of animal density in a single chamber on computing time as well as accuracy of tracking. Interestingly the increase in number of animals per chamber from 1 to 16 had only minor impact on computing time denoted by approximately 30% or no difference for Ethovision and ToxTrac, respectively (Fig. 5A). Furthermore, the quantitative endpoint such as calculated average animal velocity was not significantly affected by an increased number of tracked objects (Fig. 5B).

Next we sought to assess impact of simultaneous recording of multiple test chambers using a single camera. A constant of one organism per arena was tracked with increasing arena quantity to ensure any changes in precision were due to the change in arena quantity. As discussed in regards to resolution, the ToxTrac suite recommends the subject size per arena should be more than 50 pixels to enable for accurate thresholding and consistent results. At 18 arenas, the arena size is less than 60 pixels; beyond the recommended limit for thresholding in ToxTrac. Ethovision’s multiple arena module is advertised to be able to easily threshold and track up to 100 arenas at once, with the arena pixel quantity 200 times larger than the subject pixel area to be tracked. The ease of thresholding of Ethovision in comparison to ToxTrac was qualitatively confirmed at 18 arenas, with more than double the time taken to threshold as the organism area pixel recommendation was approached. The increase in number of arenas of interest from 1 to 18 had a minor impact on computing time denoted by 5% and 20% difference for Ethovision and ToxTrac, respectively (Fig. 5C). Furthermore the quantitative endpoints such as computed average animal velocity were not significantly affected (Fig. 5D).

The number of organisms per arena and number of arenas per video file are important variables to be considered when designing a higher throughput behavioural experiment. The impact of multiple arenas on accuracy of tracking is closely related to the resolution of the camera sensor. Multiple arenas of interest on a single video file are digitally defined in the software package. This process reduces the effective pixel quantity per area and can become a significant limitation when employing low-resolution cameras. In this work we employed a high definition 1080 p sensor and found that imaging even larger quantity of chambers might require systems equipped with ultra-high definition sensors such as 4 K (2160 p) and 8 K (4320 p) technologies. This higher resolution allows for more subjects to be within the recommended pixel subject size limit of 50 for ToxTrac with a larger amount of arenas. Unfortunately none of the animal tracking software available at present is capable of processing ultra-high definition video files desired for a high-throughput pipeline. This is a key limitation that software tracking systems are yet to overcome.

When tracking multiple small aquatic organisms contained in a single chamber it is also important to consider that they move in three-dimensional space of the chamber (Bownik, 2017). When recording objects with a single camera to achieve higher throughput of acquisition, the tracking is performed in two dimensions (2D). This means there is a potential for the algorithms to misinterpret data when organisms interact or overlap each other in 3D space (Bownik, 2017; Noldus, Spink & Tegelenbosch, 2001). Therefore, increasing the number of organisms in the test chamber can also increase the identity error of subject tracking if this benchmark is not observed. The data in Fig. 5 indicate that by employing a high definition camera and careful optimization it is possible to obtain consistent and precise results even with 16 small aquatic organisms in a single arena; with up to 18 simultaneous arenas.

### High-throughput behavioural phenotyping—batch processing

The capability to performed animal tracking using batch processing of large sets of video files is another fundamental key enabler for high-throughput behavioral phenomics. In this study only ToxTrac and Ethovision suites had a capability for batch processing of videos, however both packages differed in the implementation of this analytical feature.

The major feature of ToxTrac package was that an entire library of multiple video files could be batch imported into the software simply through sequential annotation of file names. The software had also an integrated module enabling automated pixel-to-distance calibration of all videos thus completely removing variations in calibration due to a manual user input. The disadvantages of ToxTrac in regards to batch processing were that arena and subject thresholding definitions can only be defined once. A time consuming manual review of output data was often required to ensure each subject was detected properly during the duration of each recording. Any changes required in thresholding would result in re-acquisition of the video file. Moreover, the significant spreadsheet output file providing a rather complex ensemble of numerical data was difficult to process and highlights a further need to improve analytical tools suitable for automated presentation and rapid mining of data. Because this suite is open source, it can potentially lend itself to a greater potential for customized module developments.

Compared to ToxTrac, the proprietary Ethovision XT package provides some distinct advantages. Although the import of multiple videos is not user friendly and requires significant amount of time consuming manual tasks, Ethovision features a very flexible arena definition and dynamic subject thresholding tool that can be customized to individual videos. This dynamic thresholding involves the video playing in real time as values for thresholding are changed; invisible subject tracking time and interpolated (estimated) subject tracking time are updated in real-time. This allows for accurately locking the subject pixel area in comparison to the background prior to acquisition of the entire video tracking data; thus drastically reducing the user time required to correctly identify subjects. Interestingly, this proprietary package does not have an automated pixel-to-distance calibration capability creating a potential for user error especially when dealing with large video libraries. The data analytics and graphical outputs available from Ethovision allow, on the other hand to very easily manipulate and export large data sets in a distinctly usable way. These data sets can be viewed graphically; not only with trajectory diagrams and heat maps but instantaneous movement graphs of organisms in respect to time; integral when manipulating data from sensorimotor response assays. There are additional modules available from Ethovision that allow for organism tracking to be completed in real-time with the data outputs potentially able to integrate into an organism stimulus control system. This module could open up avenues in high throughput cognitive behavioural assays.

### Benchmarking of high-throughput behavioral phenotyping in aquatic toxicology

**Figure 6:**
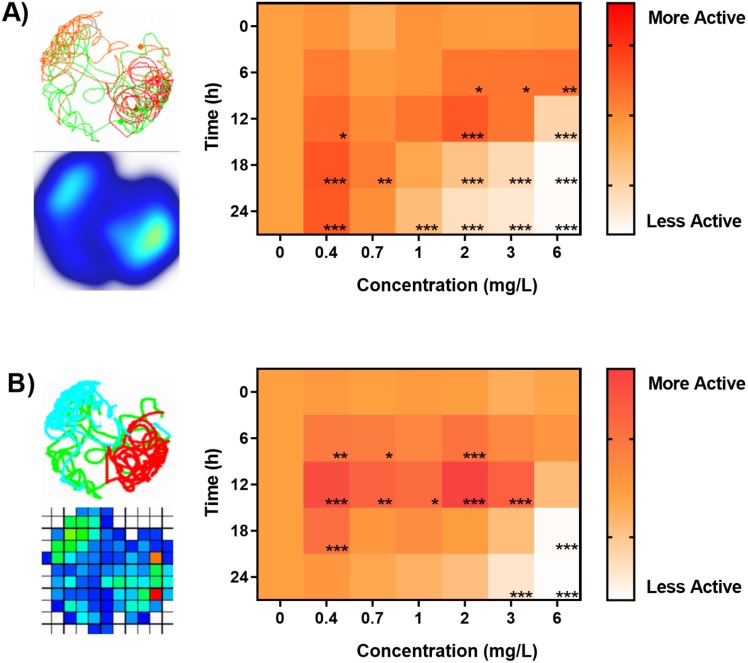
Benchmarking of animal tracking algorithms for behavioral phenotyping in toxicology. Behavioural barcoding of time-resolved data upon exposure to increasing concentrations of Chlorpyrifos as analysed by (A) Ethovision XT ver. 14 and (B) ToxTrack ver. 2.83 software suites. Left panels depict example reconstructions of graphical trajectories and occupancy map at 24 hrs and 0.4 mg/L. Right panels demonstrate a matrix heatmap analysis of a high-throughput time-resolved behavioural phenotyping screening test. All data sets were normalized to controls in each respective time-point.

**Figure 7:**
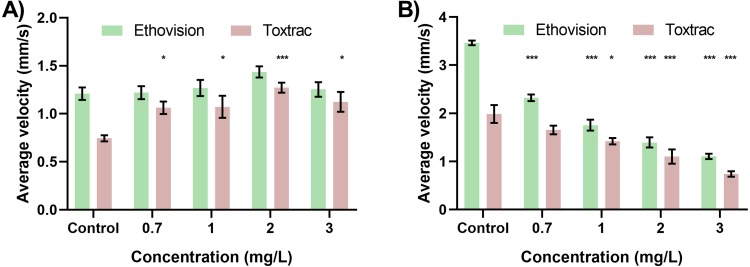
Neurobehavioural validation tests highlight hyperactivity and hypoactivity in response to varying concentrations of Chlorpyrifos. The resultant velocity of the organism (A) indicated the behavioural pattern of hyperactivity with the specified concentrations at 12 h. The resultant velocity of the organisms (B) indicated the behavioural pattern of hypoactivity with the specified concentrations at 24 h. All data sets were tested for significance relative to respective controls.

Lastly we sought to answer if analysis of identical digital video data sets performed in by different animal tracking software will have a significant impact on chemobehavioural phenotyping in aquatic toxicology. For this purpose we performed a high-throughput behavioral experiment utilizing organophosphate acetylcholine esterase inhibitor (OP; Chlorpyrifos 0 –6 mg/L) as a model aquatic toxicant. The data were analyzed in a batch-processing pipeline described above. Figure 6 demonstrates the bi-phasic responses in spontaneous locomotory activities (kinesis) of *Artemia sp.* nauplii exposed to OP as a function of time and concentration. Behavioural data revealed significant hyperactivity of nauplii upon exposure to sub-lethal concentrations of OP (Figs. 6A–6B). As described before the hyperactivity can be hypothesized as a rapid reaction to chemical stress that causes the animals attempting to escape the affected area (Faimali et al., 2017; Huang et al., 2016). The agitation of nauplii was followed by a progressive inhibition of swimming activity (hypoactivity) upon longer exposures as well as during exposures to higher concentrations of OP (Figs. 6 and 7). Overall, chemobehavioural data presented in matrix heatmaps demonstrate that analysis of identical data sets using both Ethovision XT and ToxTrac result in similar trends although certain differences can be distinguished (Fig. 6). Compared to conventional methods of summarizing behavioural data, heatmap barcodes provide one of the most convenient ways to rapidly compare results from high-throughput biometric experiments (Figs. 6 and 7). However, as shown upon statistical analysis there is a significant difference between the identical data sets analysed using both software algorithms (Figs. 6 and 7). The latter stems most likely from the inherent differences between compound errors in thresholding when implemented in automatic pipeline by different algorithms.

### Experiment design considerations for high-throughput neurobehavioral phenotyping

Neurobehavioral biometric data are sensitive and multiparameter endpoints of high physiological relevance, applicable to a plethora of toxicological and pharmacological studies. The main reservations against behavioral toxicity studies have origins in historically low reproducibility and significant heterogeneity of behavioral experiments. In this work we demonstrated that by employing careful controls as well as high-throughput behavioural analysis paradigms one can achieve robust biometric data sets. Furthermore, high-throughput behavioural analysis provides a straightforward capability to create time-resolved neurobehavioral assays with simultaneous replicates across multiple concentrations and time points.

When designing high-throughput neurobehavioural experiments using video-based tracking as the main analytical technique, it is important to anticipate all key biometric outputs that can be generated as well as any potential biasing factors. All components of the experiment design including chamber geometries and sizes, illumination sources as well as any physical stimuli (if required for e.g., sensori-motor or cognitive analysis) need to be taken into account and validated prior to the chemobehavioral assays to establish an unbiased control baseline. Furthermore, due to relatively large heterogeneity of behavioral responses among most species, all experiments run even on the same day should have their own independent sets of negative as well as validated positive controls. A particular consideration should be given to the appropriate video-recording facilities. Ideally, they should isolate the specimens from the surrounding environments to reduce any impact of external illumination, vibrations and sound-waves that can bias the behavioral biometric outputs.

In addition the video acquisition settings such as framerate, resolution, codecs and post-processing method need to be identical for any independent experiments to be quantitatively comparable. Our data also indicate that caution should be applied when comparing experiments analyzed using different animal tracking algorithms. Although global trends of biometric outputs appear to be similar, careful statistical analysis revealed significant difference between the identical outputs using different software algorithms employed. As such, the variability between different tracking algorithms should be taken into account when designing standardized behavioral experiments and conducting chemobehavioural phenotyping.

## Conclusions

The main goal of this work was to demonstrate the impact of digital video file parameters and inherent differences in proprietary and open source animal tracking software algorithms on performance and accuracy of quantitative neurobehavioural data outputs. Due to intrinsic variability of video file parameters, such as resolution, frame rate, file containers, codecs and compression levels we sought to benchmark if any of those parameters can significantly affect performance and accuracy of animal tracking algorithms. The presented data and the specific validation in toxicological chemobehavioural phenotyping demonstrate that some video parameters and techniques, such as carefully optimized video file compression and post-processing techniques can be beneficial for both increasing speed of computing and accuracy of output data. On the other hand, the transcoding of video files to lower frame rates leads to irreparable loss of data leading to biased results.

We also demonstrated impact of digital video file optimization techniques that may aid in thresholding during contrast-based animal detection. Post-processing a video file with background subtraction not only increases the accuracy of the outputted data as stated above; it also qualitatively shortens the user time in thresholding the organism against the background by twofold.

Interestingly, we found variability in certain quantitative data outputs between different animal tracking algorithms exists and data analysis performed with different software packages can be a source of significant experimental biases. We postulate that caution needs to be taken when choosing a particular animal tracking software and all data pertaining to any projects that have to be directly compared should be performed within the same software package. To the best of our knowledge, this work is so far the most comprehensive side-by-side benchmarking of most popular animal tracking software suites. Although our evaluation was performed on only one proxy biological model manifesting simple kinesis behaviors, the data nevertheless highlights potential bias between different animal tracking algorithms. More studies need to be performed in different chemobehavioural assays and more advanced functional endpoints to fully assess impact of variability in animal tracking algorithms.

Lastly, we demonstrated that higher throughput animal tracking in conjunction with automated behavioural data analysis is at present, largely underdeveloped. Although semi-automated batch animal tracking in multiple video files is possible in some software suites there is a general lack of fully integrated all-in-one solutions to perform fully automated video file transcoding, optimization, analysis and deep data mining. In line with a growing number of reports, we suggest that in combination with high-throughput automated data acquisition and analysis systems, quantitative behavioural phenotyping utilizing small model organisms can provide distinctive advantages in translational neuropharmacology, neurotoxicology and behavioral ecotoxicology (Bownik, 2017; Faimali et al., 2017; Faimali et al., 2006; Green et al., 2012; Kokel & Peterson, 2008; Zhang et al., 2019).

We postulate, however, that critical to unlock the full potential of behavioral phenomics will be high-throughput, automated data analysis and data mining utilizing potential machine learning algorithms to find trends and build predictive *in silico* models (Kokel et al., 2012; Laggner et al., 2012; McCarroll et al., 2016). At present, analytical and bioinformatics approaches that can perform rapid analysis and mining of multi-dimensional behavioural data do not exist. Automated techniques that go well beyond simple analysis of video files to reconstruct animal trajectories and obtain a set of basic behavioural parameters, such as speed of locomotion or distance travelled, will be necessary to discover discrete patterns of behavioral responses, generate new digitized behavioral barcodes, build phenoclusters and even accelerate nonlinear system-level modeling to deduce mode of action (Kokel et al., 2012; Laggner et al., 2012; McCarroll et al., 2016). However, at present even automated software techniques that can optimize and analyze hundreds of video files using object tracking and movement trajectory reconstruction in a user-friendly and high-throughput manner are largely missing. We anticipate that development of system-level multi-dimensional video analytics tools in behavioral phenomics will be paramount to many fields of research from experimental ethology, ecotoxicology to neuroactive drug discovery.

##  Supplemental Information

10.7717/peerj.7367/supp-1 Data S1 Raw Data Graphpad Prism file including all raw data to all figures.

## Additional Information and Declarations

### Data Availability

The following information was supplied regarding data availability: Raw numerical data is available as a Supplemental File. The behavioral tracking software, Toxtrac, is available for download here: https://sourceforge.net/p/toxtrac/wiki/.
